# Mathematical Framework for the Effects of Sample Absorption on Raman Intensity for Quantification and Detection Thresholds of Minerals in Two-Component Geological Mixtures

**DOI:** 10.1021/acs.analchem.6c02428

**Published:** 2026-09-22

**Authors:** Yu Yu Phua, Bethany Ehlmann

**Affiliations:** † Division of Geological and Planetary Sciences, 6469 California Institute of Technology, Pasadena, California 91106, United States; ‡ Laboratory for Atmospheric and Space Physics, University of Colorado Boulder, Boulder, Colorado 80303, United States; § Department of Geological Sciences, University of Colorado Boulder, Boulder, Colorado 80309, United States; ∥ Department of Astrophysical and Planetary Sciences, University of Colorado Boulder, Boulder, Colorado 80309, United States

## Abstract

On Earth and other planets, Raman instruments can characterize minerals *in situ* in a nondestructive and rapid manner without sample preparation. However, they commonly detect phases in mixtures because field instruments are often limited in the magnification that can be achieved (≳100 μm/spot). The effects of mixing can create challenges in detecting and quantifying the prevalence of phases of interest, particularly when there are dark minerals that attenuate photons nonlinearly with their abundance. In this work, we develop mathematical models that parameterize the effects of sample absorption on Raman intensity for three types of two-component solid mixtures: (1) low absorptivity + low absorptivity minerals, (2) low absorptivity + high absorptivity minerals, (3) high absorptivity + high absorptivity minerals. Subsequently, we verified these models through laboratory measurements of Raman peak intensities for hydrated sulfates and phyllosilicates in mixtures. We find that Raman intensity as a function of concentration is linear for mixtures made of solely low absorptivity minerals but follows a nonlinear relationship for mixtures containing one or more high absorptivity minerals. Data quantify the challenges of detection of dark minerals with Raman spectroscopy, e.g., the hydration peak of 13 vol % nontronite is detected in a low absorptivity anhydrite matrix, but three times that amount is required to detect nontronite in a high absorptivity basalt matrix. On Mars or other field settings with limited independent data, our models can be used in conjunction with microimages or other quantitative compositional instruments to estimate the detection thresholds and abundances of minerals.

## Graphical abstract

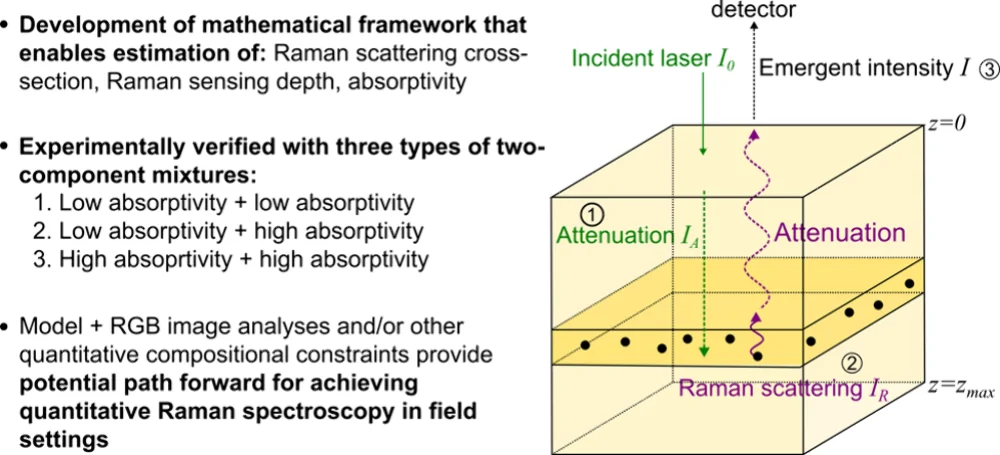

## Introduction

Raman spectroscopy is becoming increasingly common in Earth and planetary science research as it enables characterization of minerals while preserving textural relationships in rocks. Miniaturization of Raman instruments now extends applications to *in situ* field use in a non-destructive and rapid manner without any sample preparation. In addition to portable Raman instruments that are used in terrestrial settings,^1^ there are currently two Raman spectrometers operating in an extraterrestrial environment on Mars on the NASA Mars 2020 Perseverance rover: the Scanning Habitable Environments with Raman and Luminescence for Organics and Chemicals (SHERLOC) that uses a 248.6 nm laser excitation source^2^ and the SuperCam Raman spectrometer that uses a 532 nm laser excitation source.^3^ There are further planned planetary missions with a Raman spectrometer, including the Martian Moons Exploration (MMX) mission to Phobos,^4^ as well as the ExoMars mission to Mars.^5^

While the SHERLOC and SuperCam Raman spectrometers have provided valuable data on the mineralogy of Jezero crater,^6–10^ one challenge of Raman spectrometers in the field is that they are commonly measuring phases within mixtures because field instruments are often limited in the magnification that can be achieved. Benchtop Raman spectrometers are typically operated with a high magnification objective lens to focus the laser spot size to ∼few μm, which is below the typical 10s to 100s of μm grain size of the majority of mineral grains. In contrast, for commercial portable field spectrometers, the laser spot size can be as small as ∼10 μm but more typically is sub-millimeter.^11,12^ The laser spot size of SHERLOC is ∼100 μm,^2^ and that of the SuperCam Raman spectrometer is few millimeters.^3^ Consequently, with field instruments on Earth and Mars, the Raman scattered photons will originate from an area that will often contain more than one phase. This outcome has been observed with SHERLOC, where peaks originating from two different minerals are observed in the same spectrum.^7,8^

The effects of mixing can create challenges in detecting phases of interest, particularly when there are dark materials in the field of view of the laser. Here, we refer to “dark materials” as materials with large absorption coefficients in the range of the wavelength of the excitation laser and/or the Raman scattered light. Attenuation of the exciting laser and any Raman scattered photons can result in weak Raman peaks, and hence dark materials can be difficult to detect and characterize with Raman spectroscopy. We can similarly expect the Raman signal of light materials in a mixture with dark materials to be diminished as a result of attenuation by the dark materials.

Despite the importance of the effects of mixing on the detectability of minerals, few works have explored the phenomenology of this issue. Vítek et al.^13^ explored the limits of detection of organic molecules in a halite matrix using a portable Raman instrument with a laser spot size of ∼100 μm and a benchtop instrument with a laser spot size of ∼2 μm. The portable instrument performed better only at very low concentrations of 1 and 10 mg kg^–1^ β-carotene in halite, which Vítek et al.^13^ attributed to the larger spot size being better able to capture the average bulk concentration of the organics dispersed in the halite matrix. Some works have studied the Raman spectra of two- or three-component mineral mixtures, focused on the goal of constructing calibration parameters to quantify the abundances of the different components in the mixtures.^14–19^ Demaret et al.^15^ found that the intensity ratio of the gypsum to quartz Raman peaks in a gypsum–quartz mixture was not linear as a function of the weight fraction of gypsum or quartz and attributed this to the differences in the Raman scattering cross sections between gypsum and quartz. Breitman et al.^20^ studied the Raman spectra of two-component mixtures of pigment material and noted that for an anatase–rutile mixture, which are both low-absorptivity materials, the sum of the peak areas of each component is linear to the proportions in which they are present in the mixture. However, for mixtures made up of one light pigment and one dark pigment such as rutile and ultramarine blue, Breitman et al.^20^ described the relative intensities of the peaks originating from each of the components as showing “a nonlinear behaviour” that is “a complicated phenomenon” that could be a result of reflection, absorption, transmission and/or grain size.

Several works have examined the effects of absorption on the intensity of Raman peaks for an aqueous solution, whether the absorbing component is the same as the Raman-scattering component (a phenomenon known as self-absorption) or different.^21–24^ These works showed that the effects of absorption on the Raman peak intensity can be characterized by the Beer-Lambert law, which states that the emerging light decays exponentially as a function of concentration of absorbing material and distance traversed in the medium. Based on this, intuitively, we can see that the Raman peak intensity of a particular mineral will differ drastically, depending on whether it is mixed in an environment with dark, highly absorbing minerals (such as iron-bearing silicate minerals) or light, less absorbing minerals (such as evaporative salts).

Motivated by the question of detectability of minerals in a mixture with field instruments of large laser spot size but broadly applicable to any Raman system studying multi-component systems in analytical chemistry applications, in this work, we present a mathematical model that parametrizes the Raman peak intensity as a function of sample absorption and Raman cross-section for two-component solid mixtures. In geology, the effects of sample absorption are particularly important as many minerals, especially Fe-containing minerals, have electronic or crystal-field absorption features at the ultraviolet (UV), visible, or near-infrared wavelengths, all of which are commonly used laser excitation wavelengths in Raman spectroscopy. We have developed this model based on the formalisms of Hong and Asher,^21^ Lin et al.,^22^ Shriver and Dunn,^23^ and Wu et al.,^24^ where only one component is absorbing and/or Raman scattering, and extended the approaches to describe a general two-component solid mixture, where both components could be absorbing and Raman scattering at the relevant frequencies. Subsequently, we verified this model through laboratory measurements of the Raman spectra of three types of two-component mixtures, focusing on the hydration (H_2_O/OH) peaks: (1) low absorptivity + low absorptivity minerals, (2) low absorptivity + high absorptivity minerals, and (3) high absorptivity + high absorptivity minerals. We will discuss how we can use these models to estimate the quantitative values of absorptivity, Raman scattering cross section, and Raman sensing depth and how these models have the potential to provide a relatively straightforward way of developing an in situ calibration model that enables some quantitative analyses from Raman spectroscopy on Mars.

## Parameterizing the Effects of Sample Absorption and Raman Cross-Section on Raman Intensity for a Two-Component Solid Mixture

Absent other effects, the intensity of Raman peaks depends on the Raman scattering cross-section in a linear manner. The Raman intensity also depends on wavelength-dependent sample absorption, which can attenuate both the incident beam as well as the scattered Raman photons, causing the emerging light to decay exponentially as a function of concentration of absorbing material, as described by the Beer-Lambert law.^21–24^ Sample absorption can significantly affect the Raman intensity because the absorption cross-section in the ultraviolet (UV) to visible wavelength range (∼10^–16^ cm^2^ molecule^–1^ str^–1^ ^25^ is many orders of magnitude larger than the Raman cross-section (∼10^–30^ cm^2^ molecule^–1^ str^–1^).^21,25^

The effects of absorption on the intensity of Raman peaks can be understood and characterized by examining the segments of the path of the incident laser excitation light: absorption by the sample as the incident light travels through the sample (labeled ① in Figure 1), Raman scattering (labeled ② in Figure 1), and subsequent absorption by the sample of the Raman-scattered photons as they travel out of the sample into the detector (labeled ③ in Figure 1). Even though Hong and Asher,^21^ Lin et al.,^22^ and Shriver and Dunn^23^ developed their formalisms of the effects of absorption on the intensity of Raman peaks to study liquid solutions, the general optical phenomena of absorption, as described by exponential attenuation and Raman scattering, are applicable for solid mixtures as well. We also extend their formalisms to describe a general two-component solid mixture, where both components could be absorbing and Raman scattering at the relevant frequencies.

**Figure 1:**
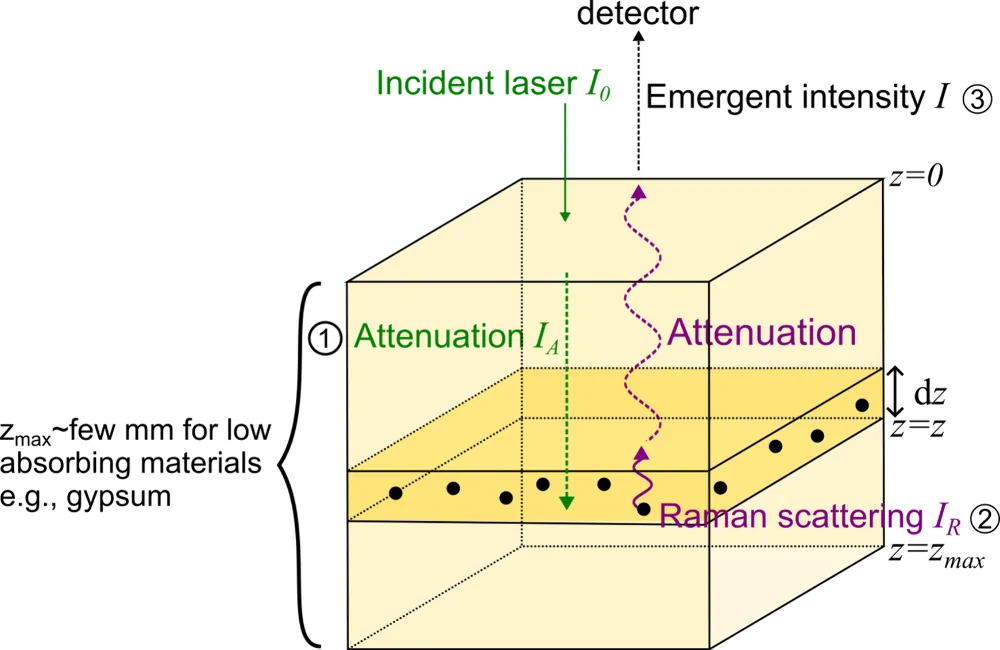
Schematic showing the path of the incident laser excitation light: ① absorption by the sample as the incident light travels through the sample (*I*_*A*_), ② Raman scattering (*I*_*R*_), and ③ subsequent absorption by the sample of the Raman-scattered photons as they travel out of the sample into the detector (*I*) for a given portion, *dz*, of the total Raman sensing depth *z*_max_. The black circles represent the Raman scattering component in this two-component mixture, and yellow the absorbing component. The scattering and absorbing components are depicted as separate materials here for illustration purposes but both materials in the mixture could be both scattering and absorbing. The sensing depth, *z*_max_, is ∼few mm for low-absorbing materials such as gypsum.^34,35^

However, one important difference between solid and liquid materials is that the propagation of the incident and Raman-scattered photons through solid materials such as the pressed pellets that we have used in this work is complicated by the multiple elastic scatterings that occur due to the inhomogeneities in the microscopic structure of the samples. In models where photon travels only in one direction, scattering is combined with absorption into an attenuation coefficient such that the intensity of light at a distance *z* is given by *I*(*z*) = *I*_0_ *e* ^–α*z*^, where *I*_0_ is the incident intensity and α = *k* + *s*, where α is the combined absorption (*k*) and scattering coefficient (*s*).^26,27^ In a two-directional flux model, Schrader et al.,^28^ based on the Kubelka-Munk theory, showed that the Raman intensity of powdered samples can be expressed as a function of the absorption and scattering coefficient. However, these models are approximations and in reality, photons can scatter in any direction in three dimensions in a random-walk manner.

The effects of scattering on Raman intensity are dependent not only on sample characteristics such as crystal orientation and grain sizes but also the optics of the instrument. Smaller grain sizes are expected to increase the amount of multiple scattering. However, whether multiple scattering leads to enhanced or attenuated Raman intensity depends also on the laser beam size used. The exponential attenuation model that combines scattering into the attenuation coefficient and the Kubelka-Munk theory predict that increased elastic scattering will result in decreased Raman intensity.^28^ This trend is observed for small laser beam sizes (<5 μm) with graphite and coal samples.^29^ However, for instruments with a larger laser beam size (>100 μm), multiple scattering can lead to the excitation of more Raman-active components within the volume that is probed by the laser, resulting in an increase in Raman intensity that is not consistent with theoretical predictions.^30^ The competing effects of multiple scattering due to the instrument optics and grain size appear to result in a non-monotonic trend of Raman intensity as a function of grain size. Specifically, previous works have found that the Raman intensity is maximized at an intermediate grain size (∼30–100 μm) for olivine, feldspar and carbonates but pyroxene does not show a consistent trend.^31–33^ Indelicato et al.^33^ observed an increase in Raman intensity as a function of grain size from 10–50 μm for calcite, with the Raman intensity at 50 μm ten times greater than at 10 μm. They modeled this increase with the formulation of Schrader et al.^28^ based on the Kubelka-Munk theory, incorporating the thickness of the Raman-scattering layer, Raman scattering cross-section, absorption coefficient, and scattering coefficient.^33^ For grain size 50–900 μm, the Raman intensity of calcite was observed to decrease as a function of grain size, with the Raman intensity at 900 μm half of that at 50 μm. They modeled this decrease with the reflectivity of the air–particle interface and the absorption coefficient.^33^

Due to the nontrivial and not fully studied nature of the effects of grain sizes and multiple scattering on Raman intensity, in this work, we make a simplifying assumption that the contributions of elastic scattering are small compared to absorption. This is not a bad assumption for the pressed pellet samples that we have used, which are expected to have few pore spaces and hence less scattering^34^ than powdered samples studied in previous works.^28,30–33^ We verified the validity of this assumption in the “Experimental Verification of Model As Relevant to Geologic Materials” section through laboratory measurements where the grain sizes are not controlled and the laser spot size is relatively large (∼30 μm). We emphasize that quantitative analyses of Raman spectroscopy for solid samples including pressed pellet samples will benefit from a future more sophisticated treatment of the elastic scattering that arises from the microscopic heterogeneity of solid materials. Considerations of multiple scattering in the model will affect the measured Raman sensing depth, though our estimates with this simplified model predict results to within an order-of-magnitude or better (see “How Mathematical Relationships Inform Absorptivity of High Absorptivity Materials and Raman Sensing Depths of Low Absorptivity Materials”).

We are interested in modeling the Raman scattering contributions from both components because, in some of our experimentally studied samples (see “Experimental Verification of Model As Relevant to Geologic Materials”), the hydration peak can originate from multiple minerals. The hydration peak at ∼3000–4000 cm^–1^ is caused by the O–H symmetric and asymmetric stretching of both molecular H_2_O and structural hydroxide OH and can originate from many mineral groups, including hydrous salts like sulfates and carbonates and silicates like phyllosilicates.^36–38^ In the following, we elaborate on the processes and relevant equations following the path of the incident laser excitation light, valid for a backscattering geometry, a very common geometry employed for Raman spectrometers.

Based on the exponential attenuation, for a two-component solid mixture *x* and *y*, the intensity of the incident laser at depth *z* from the surface, *I*_*A*_ (labeled ① in Figure 1), is

1 $$I_{A}=I_{0}e^{-\left(\varepsilon_{0,x}C_{x}+\varepsilon_{0,y}C_{y}\right)z}$$

where *I*_0_ is the intensity of the incident laser, ε_0,*x*_ and ε_0,*y*_ are the absorptivity of components *x* and *y* at the frequency of the incident laser with SI-units of L mol^–1^ m^–1^, respectively, and *C*_*x*_ and *C*_*y*_ are the concentrations of components *x* and *y* with SI-units of mol L^–1^. The total Raman-scattered intensity by components *x* and *y* within thickness d*z*, *I*_*R*_ (labeled ② in Figure 1), is

2 $$I_{R}={I_{R,x}+I_{R,y}=I}_{A}\sigma_{x}C_{x}dz+I_{A}\sigma_{y}C_{y}dz$$

where *I*_*R*,*x*_ and *I*_*R*,*y*_ are the Raman-scattered intensity by components *x* and *y* within thickness d*z*, and σ_*x*_ and σ_*y*_ are the Raman scattering cross sections for a particular Raman peak for components *x* and *y*, respectively. The intensity of the Raman scattered light that then travels through the sample and reaches the surface into the detector, *I* (labeled ③ in Figure 1), is

3 $$I=I_{R}e^{-\left(\varepsilon_{R,x}C_{x}+\varepsilon_{R,y}C_{y}\right)z}$$

where ε_*R*,*x*_ and ε_*R*,*y*_ are the absorptivity of components *x* and *y* at the frequency of the Raman-scattered light, respectively.

For a Raman sensing depth *z*_max_, which we define to be the maximum depth contributing to the observed Raman signal, we can get the Raman intensity, *I*, after all of the interactions by substituting eqs 1 and 2 into eq 3, and integrating from *z* = 0 to *z* = *z*_max_:

4 $$I=\frac{I_{0}\left(\sigma_{x}C_{x}+\sigma_{y}C_{y}\right)\left(1-e^{-z_{\max}\left({(\varepsilon_{0,x}+\varepsilon }_{R,x})C_{x}+{(\varepsilon_{0,y}+\varepsilon }_{R,y})C_{y}\right)}\right)}{{(\varepsilon_{0,x}+\varepsilon }_{R,x})C_{x}+{(\varepsilon_{0,y}+\varepsilon }_{R,y})C_{y}}$$

The maximum sensed depth, *z*_max_, likely depends on instrument factors such as the numerical aperture of the objective lens, as well as sample characteristics such as crystal orientation and grain sizes (see also above discussion and “How Mathematical Relationships Inform Absorptivity of High Absorptivity Materials and Raman Sensing Depths of Low Absorptivity Materials”). From eq 4, we consider two limiting cases, the first where the absorptivity of both components *x* and *y* at both frequencies of the incident laser and Raman scattered light are small. In this case, 1 – *e* ^–*z*_max_((ε_0,*x*_+ε_*R*,*x*_)*C*_*x*_+(ε_0,*y*_+ε_*R*,*y*_)*C*_*y*_)^ ≈ *z*_max_((ε_0,*x*_ + ε_*R*,*x*_)*C*_*x*_ + (ε_0,*y*_ + ε_*R*,*y*_)*C*_*y*_) and eq 4 becomes

5 $$I=I_{0}\left(\sigma_{x}C_{x}+\sigma_{y}C_{y}\right)z_{\max}$$

When instead the absorptivity of either component *x* or *y* is large, 1 – *e* ^–*z*_max_((ε_0,*x*_ + ε_*R*,*x*_)*C*_*x*_+(ε_0,*y*_+ε_*R*,*y*_)*C*_*y*_)^ ≈ 1 and eq 4 becomes

6 $$I=\frac{I_{0}\left(\sigma_{x}C_{x}+\sigma_{y}C_{y}\right)}{{(\varepsilon_{0,x}+\varepsilon }_{R,x})C_{x}+{(\varepsilon_{0,y}+\varepsilon }_{R,y})C_{y}}$$

In the following sections, we will use these two limiting cases to guide our selection of samples to verify the use of eqs 5 and 6 as relevant to geologic materials.

## Experimental Verification of Model As Relevant to Geologic Materials

### Endmember Sample Selection and Sample Preparation

To characterize how the intensities of the Raman peaks depend on the concentration of materials in mixtures of different absorptivities and different Raman scattering cross sections, our sample suite includes samples with qualitatively high and low absorptivity, as well as high and low Raman scattering cross sections. Note that there are few quantitative databases of absorptivity and Raman cross section, and these can vary in ways specific to a particular sample (e.g., due to crystallinity, minor/trace elements), so our knowledge of these parameters is qualitative/comparative only.

The specific samples we have chosen include hydrated, hydroxylated and anhydrous minerals that have either been detected on Mars or proposed to be present (Table 1). Natural samples of a basalt (K1919), the Fe-bearing phyllosilicate nontronite (NAu-2), serpentine and gypsum were prepared by Thomas et al.^39^ as pressed pellets from powdered samples of <250 μm. We made additional pressed pellets with synthetic powder samples of anhydrite, gypsum, kieserite and brucite, which were purchased from chemical supply companies and used as is, without further independent characterizations. The identities of these synthetic samples were confirmed through the Raman spectra we collected (Figure 2).

**Table 1:** List of All Endmember Samples

				characteristic	
sample	chemical formula	sample origin	sample source	absorptivity	density (g cm^–3^)
K1919 basalt^a^	n/a, polymineralic, fine-grained basaltic rock	Near USGS BHVO-1 standard locality in Kilauea, Hawaii	Caltech collection	high	3.00
NAu-2 nontronite^a^	(Ca,Na,K)_0.72_(Si_7.55_Al_0.45_) (Fe_3.83_Mg_0.05_)O_20_(OH)_4_	South Australia	ref 40	high	2.30
Serpentine^a^	(Mg,Fe)_3_Si_2_O_5_(OH)_4_	Hoboken, NJ	Caltech mineral collection	high	2.50
Gypsum^a^	CaSO_4_·2H_2_O	Fremont County, Colorado	Ward’s	low	2.30
Gypsum	CaSO_4_·2H_2_O	Synthetic, reagent grade	Sigma-Aldrich	low	2.30
Anhydrite	CaSO_4_	Synthetic, reagent grade	Sigma-Aldrich	low	2.97
Kieserite	MgSO_4_·H_2_O	Synthetic, reagent grade	Sigma-Aldrich	low	2.57
Brucite	Mg(OH)_2_	Synthetic, reagent grade	Sigma-Aldrich	low	2.39

a Samples as prepared in Thomas et al.^39^

**Figure 2:**
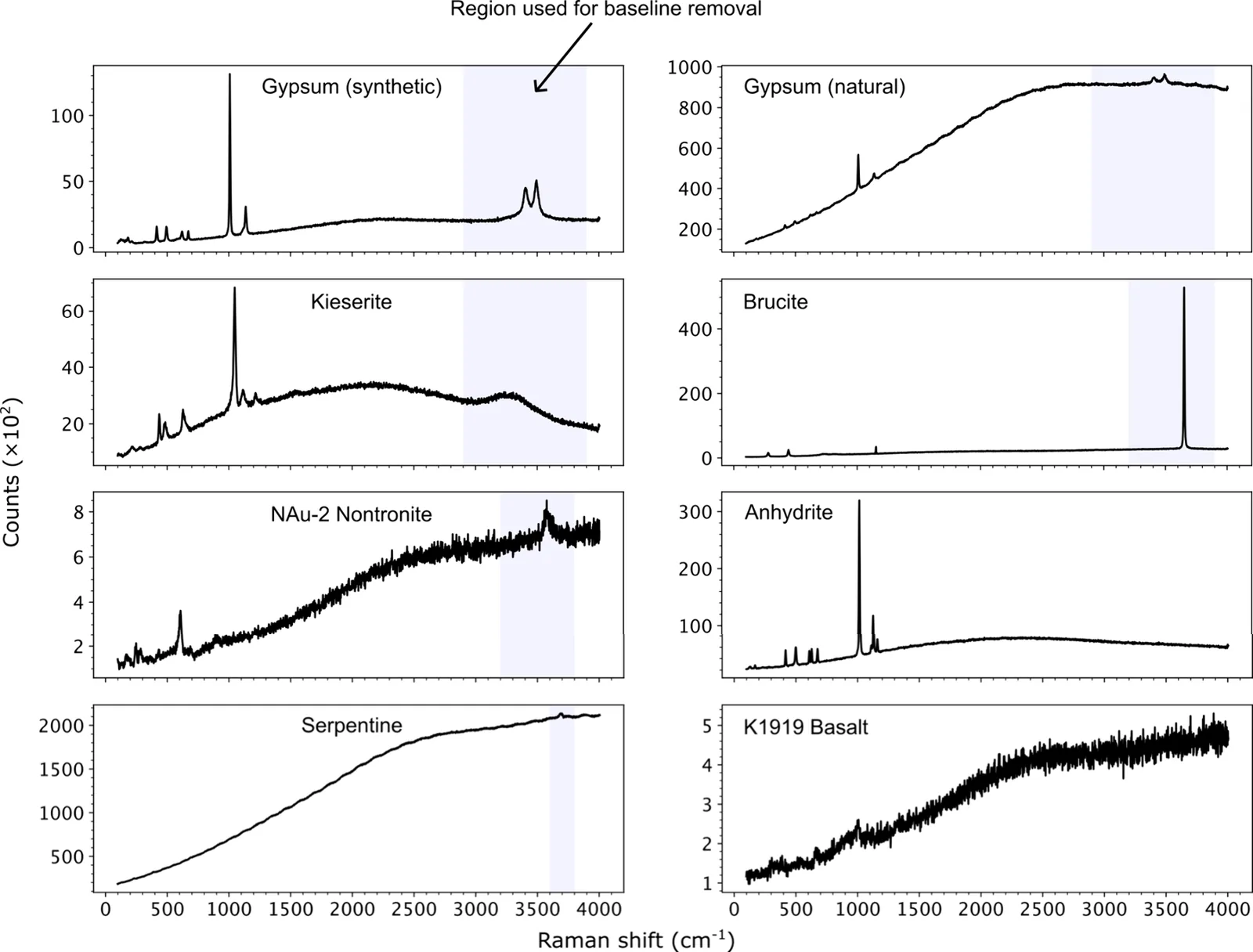
Example spectra of the endmember minerals used in this work. The shaded region indicates the region used for baseline removal for the different minerals. When hydration peaks from more than one mineral are present, the broader range is used for baseline removal (see “Acquisition and Processing of Raman Spectra”).

We mixed these different endmember powder samples at various proportions to produce three types of binary mixtures with different combinations of absorptivity: (1) Low absorptivity + low absorptivity, (2) Low absorptivity + high absorptivity, (3) High absorptivity + high absorptivity (Table 2). To create these mixtures, first we weighed out the masses of the powder samples using an analytical balance (±0.1 mg) at the relative proportions listed in Table 2. We mixed these two components in a mortar and pestle and pressed them into a 1.2 cm diameter pellet at a pressure of 124 MPa for 20 s. The thickness of the pellets was not controlled but the average thickness of 20 randomly measured pellets was 1.11 ± 0.26 mm.

**Table 2:** Types of Mixtures Prepared and Their Mixing Ratio

sample	mixing ratio (wt %)
Type 1: Low Absorptivity + Low Absorptivity	
Kieserite: Anhydrite	20:80, 50:50, 80:20
Gypsum: Brucite	10:90, 30:70, 50:50, 70:30
Type 2a: Low Absorptivity + High Absorptivity	
Gypsum: Basalt^a^	5:95, 10:90, 30:70, 50:50, 70:30
Anhydrite: NAu-2	80:20, 40:60, 50:50, 60:40, 70:30, 90:10, 95:5, 99:1
Kieserite: NAu-2	20:80, 60:40, 90:10, 95:5, 99:1
Type 2b: High Absorptivity + High Absorptivity	
NAu-2: Basalt^a^	10:90, 30:70, 50:50, 70:30
Serpentine: Basalt^a^	30:70, 50:50, 70:30

a Mixture pellets were prepared by Thomas et al.^39^

### Acquisition and Processing of Raman Spectra

We used a Renishaw Raman inVia Spectrometer with a 514.5 nm excitation source and 1,800 lines/mm grating to acquire Raman spectra from 100–4000 cm^–1^ at every 1 cm^–1^ interval. The laser power was attenuated to 10%, equivalent to 10 mW. The wavelength was calibrated with a silicon chip to within 0.1 cm^–1^. A 5× objective was used for all the mixtures except the serpentine + basalt mixture. The resulting laser beam size with a 5× objective is ∼30 μm and spectral resolution 6 cm^–1^. Even though a lower objective lens results in a lower signal-to-noise ratio (SNR), we used this to enable mixed phases to be probed in the field of view of the laser. For the serpentine + basalt mixture, we used a 20× objective, giving a laser beam size of ∼10 μm, to increase the SNR to get usable Raman spectra from the weakly scattering serpentine.

No pre-treating was conducted for all but the natural gypsum sample from Fremont County, Colorado, which we pre-treated with 150 s of photobleaching before acquiring Raman spectra to reduce the fluorescence background. For all samples but the serpentine-basalt mixtures and serpentine endmember, we collected a two-dimensional map of Raman spectra consisting of 100 points in a 10 × 10 grid covering a 1 mm × 1 mm area of the sample. The measurements were recorded using 10 s exposure time and one spectral accumulation. For the serpentine-basalt mixtures, the Raman spectral map consisted of 100 points in a 10 × 10 grid covering a 0.1 mm × 0.1 mm area of the sample. The measurements were recorded using 10 s exposure time and two spectral accumulations. For the serpentine endmember, we attempted to make measurements from hundreds of different points on the sample using 10 s exposure time and two spectral accumulations. However, the acquisition of a Raman spectrum was hindered by fluorescence from the natural serpentine sample and we were only able to successfully acquire one usable Raman spectrum of endmember serpentine.

The acquired spectra were processed with self-written Python scripts. We focused our analyses on the hydration peaks and removed the baseline locally in the range within 2900–3900 cm^–1^ in the vicinity of the hydration peaks (see Figure 2 for the specific wavenumber range for the different minerals), using a linear function to model the baseline to offset the effects of fluorescence except for the gypsum-brucite sample, as was done in Phua et al.^8^ Subsequently, we fit the hydration peaks with up to two Gaussian components for all samples except the gypsum-brucite sample, using the SciPy library of Python.^41^ The hydration peaks can be approximated with Gaussian profiles, as the intrinsic peaks for the O–H symmetric and asymmetric stretch modes are inhomogeneously broadened as a result of diverse microenvironments.^42,43^ For the gypsum-brucite sample, we modeled the baseline with the improved asymmetrically reweighted penalized least squares method using the pybaselines library of Python.^44^ In addition, we find that while the hydration peaks of gypsum from molecular H_2_O are well approximated as Gaussian functions, the narrow hydration peaks of brucite from structural OH (FWHM ∼ 7) are better approximated as Lorentzian functions. This difference is likely because the structural OH in brucite are arranged in a well-ordered crystalline lattice, resulting in a homogeneously broadened peak that is Lorentzian.^45,46^

We filtered and removed fitted peaks that are of lower confidence in subsequent analyses based on the SNR. For all samples except the serpentine + basalt samples, we calculated the SNR, which we defined to be

$$\mathrm{SNR}=\frac{\mathrm{intensity of the fitted peak}}{\mathrm{standard deviation of noise within}2000\mathrm{to}2100{\mathrm{cm}}^{-1}}$$

We calculated the standard deviation of noise within 2000–2100 cm^–1^, as no Raman peaks are present in that region. The serpentine-basalt samples had significant oscillations in the spectra due to interference fringes caused by the thin-film layers that make up the edge filter in the optics of the Raman spectroscopic system that we speculate were more readily apparent due to the low signal. These oscillations were removed using the Fast Fourier Transform algorithm. The standard deviation of noise was then calculated within 2000–2050 cm^–1^. The detection threshold was set as SNR > 2, which corresponds to >95% certainty that a peak is produced by actual signal. While SNR > 3 is a commonly used detection threshold, we chose SNR > 2 in this work to retain the hydration peaks of kieserite that are broad (FWHM > 300) but have low intensity as real detections. The implications of the chosen SNR detection threshold are discussed in “Observed Detection Thresholds and the Dominant Effect of High Absorptivity Materials”.

We used the area of the hydration peak that was retrieved from the peak-fitting process to assess the change in Raman signal strength as a function of concentration, detailed in “Results and Discussion”.

## Results and Discussion

The representative spectra of gypsum-basalt mixtures, a low absorptivity + high absorptivity suite, show that the peak intensities of the SO_4_ symmetric and asymmetric stretch, as well as OH stretch, all of which can be attributed to gypsum, increase with increasing concentration of gypsum from 30 to 100 wt %, as expected (Figure 3(A)). These spectra were chosen because they have OH peak areas that are comparable to the average OH areas calculated for the respective samples. However, there is actually substantial heterogeneity in the peak intensity spot-to-spot. The optical microscope image of the scan area for 50 wt % gypsum-basalt illustrates spatial heterogeneities in the distribution of gypsum and basalt within the sample (Figure 3(B)). Even though we have used a low magnification objective lens (5×) to increase the laser spot size, the heterogeneity of the sample is sometimes at a larger scale (>100 μm) than the laser spot size (∼30 μm), also expected given the particle size distribution of the mixtures. The heterogeneities in the distribution of gypsum and basalt correlate to heterogeneities in the Raman spectra recorded, whereby the highest OH peak areas are correlated to locations with higher concentrations of white material that is presumably gypsum (Figure 3(B)).

**Figure 3:**
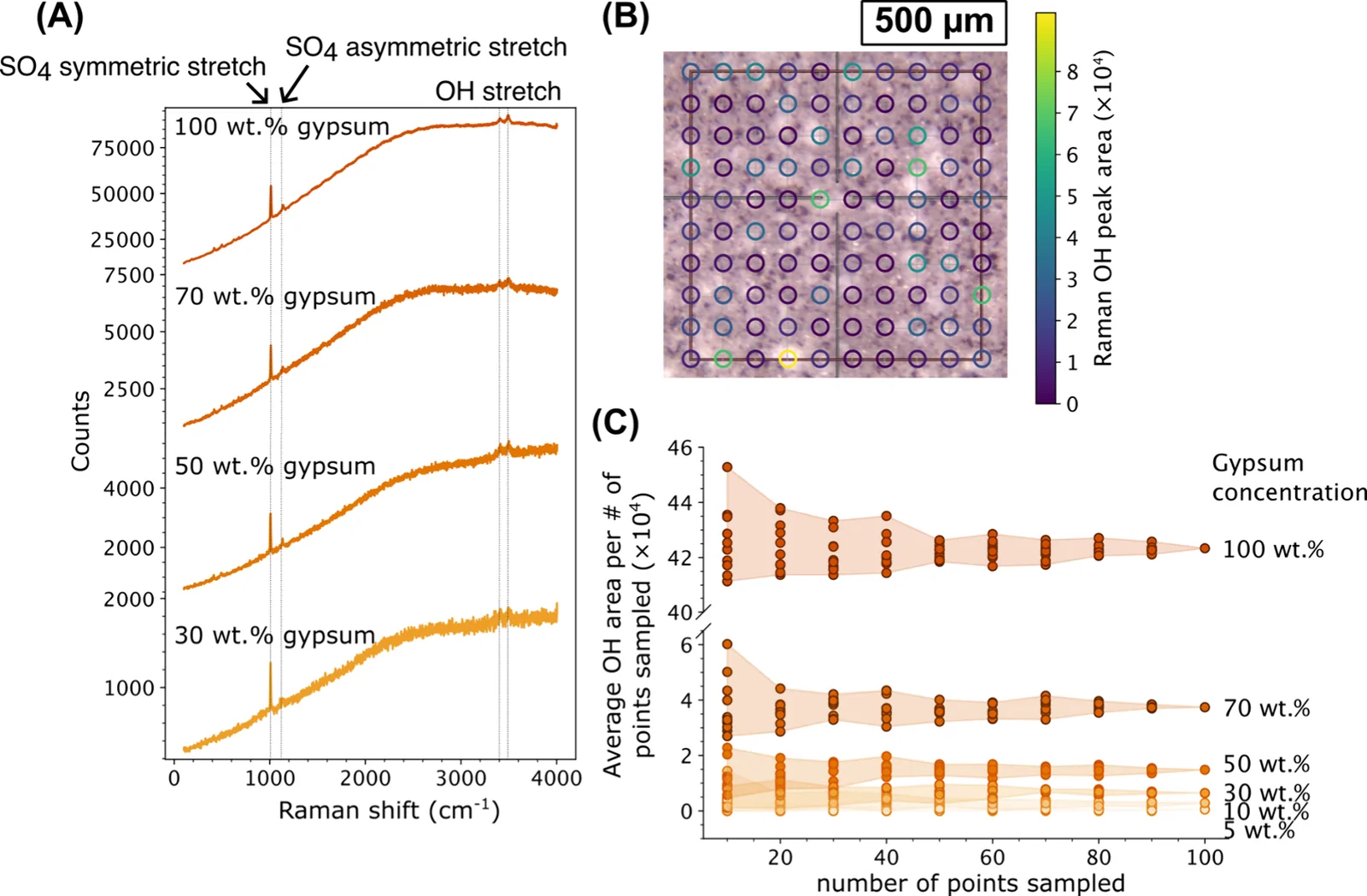
(A) Representative spectra for points from 30, 50, 70, 100 wt % gypsum-basalt, a low absorptivity + high absorptivity mixture, which have OH peak areas that are comparable to the average OH areas calculated for the respective samples. Peaks corresponding to the SO_4_ symmetric and asymmetric stretch, as well as OH stretch are labeled. (B) The circles represent the locations of measurements overlain on an optical image of 50 wt % gypsum-basalt. The color of the circles is scaled to the OH peak area at that point. Note that the size of the circles is optimized for visualization, and the actual size of the laser spot is smaller. (C) The average OH area of 10 to 90 randomly selected points was calculated and plotted as a function of number of sampled points for 5, 10, 30, 50, 70 wt % gypsum-basalt mixtures. For each number of points sampled, 10 different sets of points were selected and its average OH area calculated. The shaded area is for easy visualization of the convergence of the average area as the number of sampled points increases.

While the compositional heterogeneity at the microscopic scale of the gypsum-basalt mixture gives rise to variable OH peak areas at each measured point (Figure 3(B)), the average OH peak areas converge as the number of sampled points increases (from *n* = 10 to *n* = 100; Figure 3(C)). Mixtures with a higher concentration of gypsum converge to a higher average OH peak area, consistent with our expectations. All mixture samples showed that the average OH peak areas converge as the number of sampled points increases (Figure 4). However, for the nontronite-anhydrite samples, the average OH peak area for 50 wt % nontronite converged to a value that is larger than that of 80 wt % nontronite by ∼8%.

**Figure 4:**
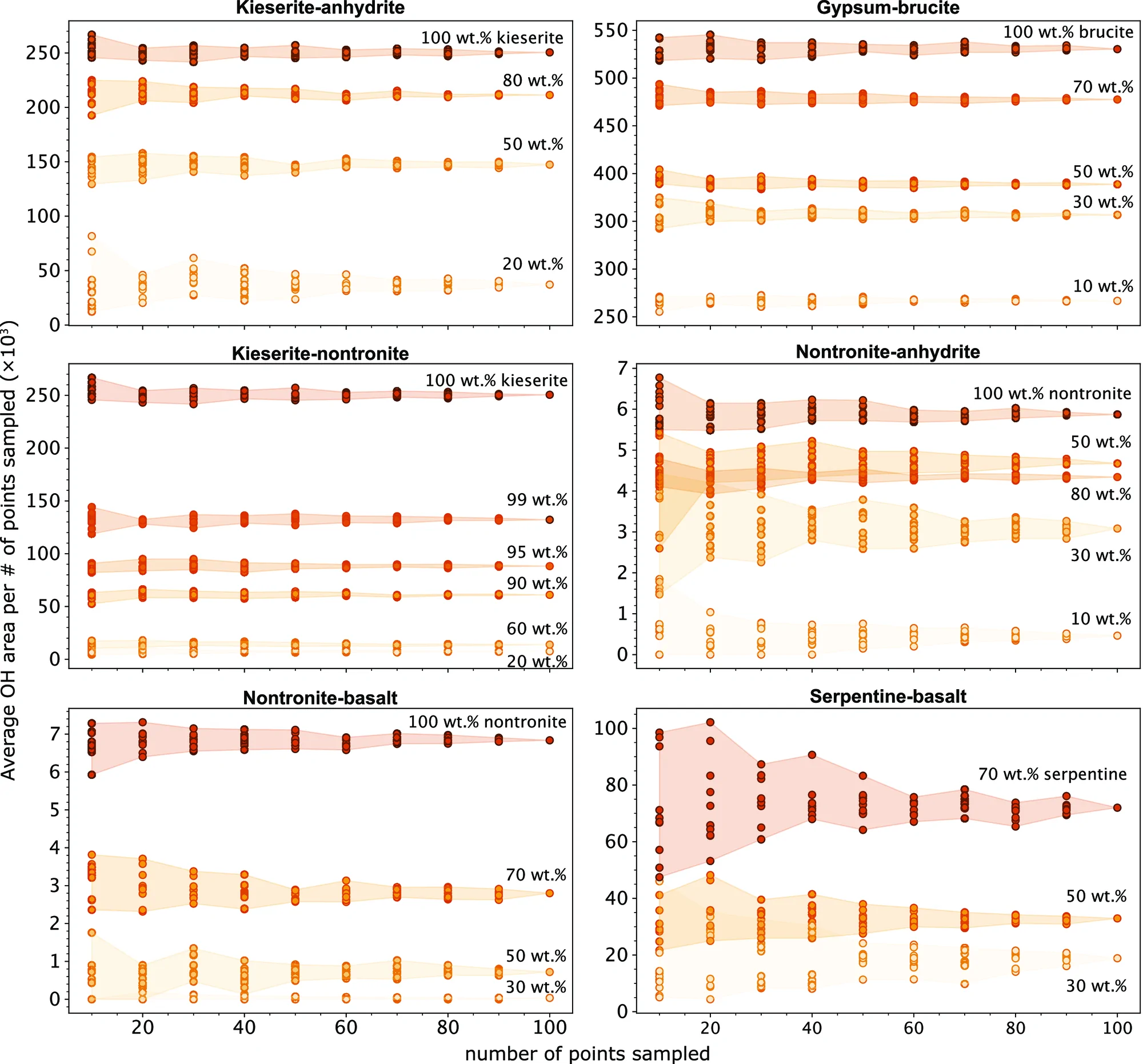
Average OH area of 10 to 90 randomly selected points was calculated and plotted as a function of number of sampled points for kieserite-anhydrite, gypsum-brucite, kieserite-nontronite, nontronite-anhydrite, nontronite-basalt, and serpentine-basalt. For each number of points sampled, 10 different sets of points were selected and its average OH area calculated. The shaded area is for easy visualization of the convergence of the average area as the number of sampled points increases. The concentration of each series is labeled in the plot, expressed as wt % relative to the endmember mineral specified in the plot.

These results show that acquiring an average of 100 sampled points over a defined region can bridge the gap between the microscopic compositional heterogeneity that is measured by Raman spectroscopy and the macroscopic bulk composition of the mixture samples. This is consistent with the results of Haskin et al.,^47^ who found that it is possible to quantify the mineral proportions in a rock sample by acquiring 100 Raman spectra from the sample and examining the proportion of spectra that contain the mineral peak of interest. Consequently, we use the average OH peak area as representative of the Raman signal intensity for each mixture sample, to investigate how the Raman intensity changes as a function of concentration and verify eqs 5 and 6.

The fit parameters for the models are derived from least-squares fitting. To quantify the uncertainties of the fit parameters, we used the nonparametric bootstrap method. Specifically, for a given mixture series, for each concentration value, we randomly sampled 100 values with replacement from our measured Raman OH peak areas to form a “new” set of Raman OH peak areas. We computed the bootstrap mean for this new set and fitted our model using the bootstrap mean at each corresponding concentration. We repeated this procedure 2000 times for each concentration value in a mixture series. The fit parameters we report here are the mean from the 2000 fits conducted, while the uncertainty is the standard deviation from the 2000 fits.

An effect of note, apparent for mixtures containing high-absorptivity materials (see “Low Absorptivity + High Absorptivity Mixtures” and “High Absorptivity + High Absorptivity Mixtures”), is a dominance of points with low signal. Approximately half the points in the 50 wt % gypsum-basalt sample record very small OH peak areas <10000 (48/100 points), with the remaining half of the points having OH peak areas of 10000–94114 (Figure 3(B)). This asymmetrical distribution of peak intensities will cause the sample’s average OH peak area to be weighted more strongly to low values closer to 0 than the arithmetic average between the maximum OH peak area (=94114) and the minimum OH peak area (=0). A similar phenomenon of asymmetrical distribution of peak intensities is also observed for low absorptivity + low absorptivity mixtures such as kieserite-anhydrite due to points that consist of purely anhydrite contributing no OH peaks (Figure 5).

**Figure 5:**
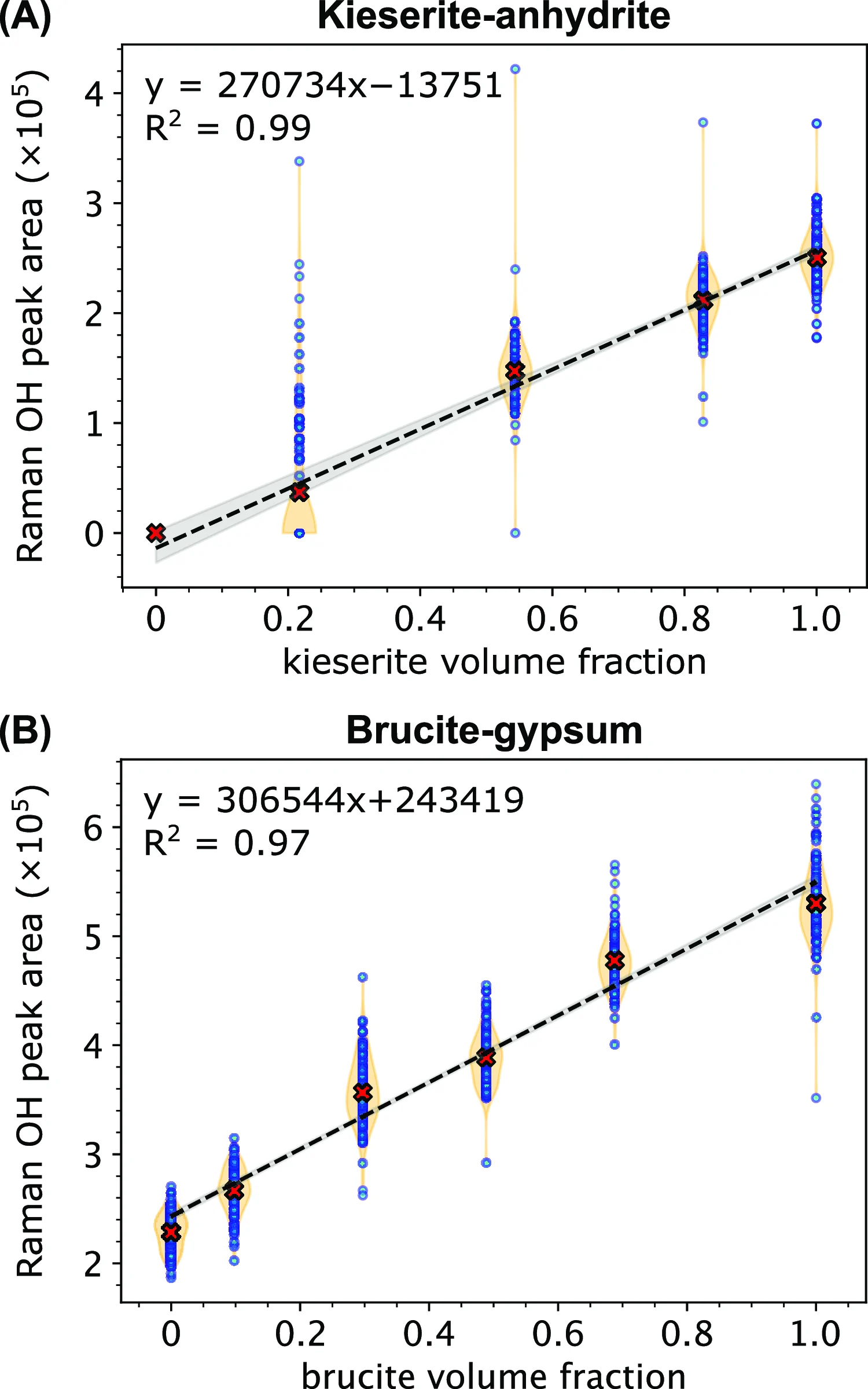
Raman OH peak area as a function of concentration of the hydrated component for two types of low absorptivity + low absorptivity mixtures: (A) kieserite-anhydrite pressed pellets, (B) brucite-gypsum pressed pellets. The circles are the individual measurement points while the red crosses are the averages of those 100 measured points for each concentration, overlain on the violin plots showing the distribution of the measured OH area for each concentration. The dashed lines are the linear trendlines fitted to the average OH area. The transparency of the points is proportional to the density of points. The gray shaded region is the 2σ-confidence interval of the model fit.

### Low Absorptivity + Low Absorptivity Mixtures

We investigated two types of mixtures for the low absorptivity + low absorptivity mixtures, for which the Raman intensity can be characterized by eq 5: kieserite-anhydrite and gypsum-brucite. Consistent with predictions from our theoretical model described in eq 5 with further simplifications detailed below in eq 10, the average OH peak areas of kieserite-anhydrite and gypsum-brucite follow a linear model as a function of concentration of the hydration component, which are kieserite and gypsum, respectively (Figure 5). While kieserite is the only hydrated component in the kieserite-anhydrite mixture, in the gypsum-brucite mixture, both gypsum and brucite are hydrated and contribute to the OH peak area.

In eq 5, *C*_*x*_ is given in units of moles per unit volume, which is the most relevant quantity for the physical interaction of the particles with light. To relate the units for our prepared mixtures by mass to moles per unit volume, we first express the concentration of our mixtures in vol %, *C*_*x*,vol_, using the mass and density of each component, *m*_*x*_, *m*_*y*_ and ρ_*x*_, ρ_*y*_, respectively:

7 $$C_{x,\mathrm{vol}}=\frac{\frac{m_{x}}{\rho_{x}}}{\frac{m_{x}}{\rho_{x}}+\frac{m_{y}}{\rho_{y}}}$$

Subsequently, we can relate *C*_*x*,vol_ to *C*_*x*_ in moles per unit volume as follows:

8 $$C_{x}=\frac{{\mathrm{mol}}_{x}}{{\mathrm{Vol}}_{t}}=\frac{\frac{m_{x}}{a_{x}}}{\frac{m_{x}}{\rho_{x}}+\frac{m_{y}}{\rho_{y}}}=\frac{\rho_{x}}{a_{x}}\times C_{x,\mathrm{vol}}$$

where mol_*x*_ and *a*_*x*_ are the number of moles and the molar mass of component *x*, respectively, and Vol_*t*_ is the total volume of the sample. Similarly, $C_{y}=\frac{\rho_{y}}{a_{y}}\times C_{y,\mathrm{vol}}$

For a two-component low absorptivity + low absorptivity mixture, eq 5 can thus be further simplified using the fact that the concentrations sum to 1, *C*_*x*,vol_ + *C*_*y*,vol_ = 1, such that the relation is linear with concentration of one of the components, e.g., *C*_*x*,vol_ below:

9 $$I=I_{0}z_{\max}\left(\sigma_{x}\frac{\rho_{x}}{a_{x}}-\sigma_{y}\frac{\rho_{y}}{a_{y}}\right)C_{x,\mathrm{vol}}+I_{0}z_{\max}\sigma_{y}\frac{\rho_{y}}{a_{y}}$$

The terms $I_{0}z_{\max}\left(\sigma_{x}\frac{\rho_{x}}{a_{x}}-\sigma_{y}\frac{\rho_{y}}{a_{y}}\right)$ and $I_{0}z_{\max}\sigma_{y}\frac{\rho_{y}}{a_{y}}$ in eq 9 can be approximated as constants A and B, respectively, and Raman intensity is thus a linear function of the concentration of component *x*:

10 $$I=AC_{x,\mathrm{vol}}+B$$

where *C*_*x*,vol_ now has units of vol %. Note that the Raman sensing depth, *z*_max_, could vary with mixture proportions if the absorptivities of the mixed materials were substantially different, but this effect is likely negligible for low absorptivity materials.

### Low Absorptivity + High Absorptivity Mixtures

We investigated five types of mixtures for the low absorptivity + high absorptivity mixtures, for which the Raman intensity can be characterized by eq 6. Of these, we have measured three mixtures, two of which contain only one hydrated component: (1) gypsum-basalt, where the hydrated component gypsum has lower absorptivity than the nominally anhydrous component basalt (contains 0.12 wt % water^39^), and (2) nontronite-anhydrite, where the hydrated component nontronite has a higher absorptivity than the anhydrous component anhydrite. For the third mixture, kieserite-nontronite, both components are hydrated, and kieserite has an absorptivity lower than that of nontronite. We also examined the general applicability of our models by using reported sulfate peak data from Haney et al.^48^ for two powdered mixtures, gypsum-palagonite and gypsum-hematite. These samples were measured with the Analogue Complementary Raman for Operations oN Mars (ACRONM) instrument at the NASA Johnson Space Center in the ARES Raman Laboratory. ACRONM is an analogue instrument for the SHERLOC Raman spectrometer on Mars 2020 and uses a 248.6 nm deep-ultraviolet laser.^7^ Palagonite and hematite contain Fe^2+^ and/or Fe^3+^, which are highly absorbing at the laser wavelength of the ACRONM. Therefore, both gypsum-palagonite and gypsum-hematite mixtures measured by Haney et al.^48^ are classified as low absorptivity + high absorptivity mixtures. We digitize and convert their reported concentration in wt % to vol %, assuming the powdered samples have 30% porosity. A higher porosity would result in an increase in total volume and decrease in gypsum volume fraction, but the volume of gypsum would remain constant. Consequently, the porosity value does not directly affect the nonlinear functional dependence of the Raman intensity on the gypsum concentration. The effect of porosity on the functional form of the model is detailed below (eq 15).

Measurements show that the average OH peak area increases with increasing concentration of the hydrated component for the gypsum-basalt, kieserite-nontronite, and nontronite-anhydrite mixtures that we have measured, albeit with different functional shapes and mathematical forms (Figure 6). That the average OH area of nontronite is close to two orders of magnitude smaller than the OH area in gypsum and kieserite, though they have similar water contents, highlights that it will generally be more challenging to characterize high absorptivity phyllosilicates such as nontronite with Raman spectroscopy compared to low absorptivity sulfates such as gypsum and kieserite.

**Figure 6:**
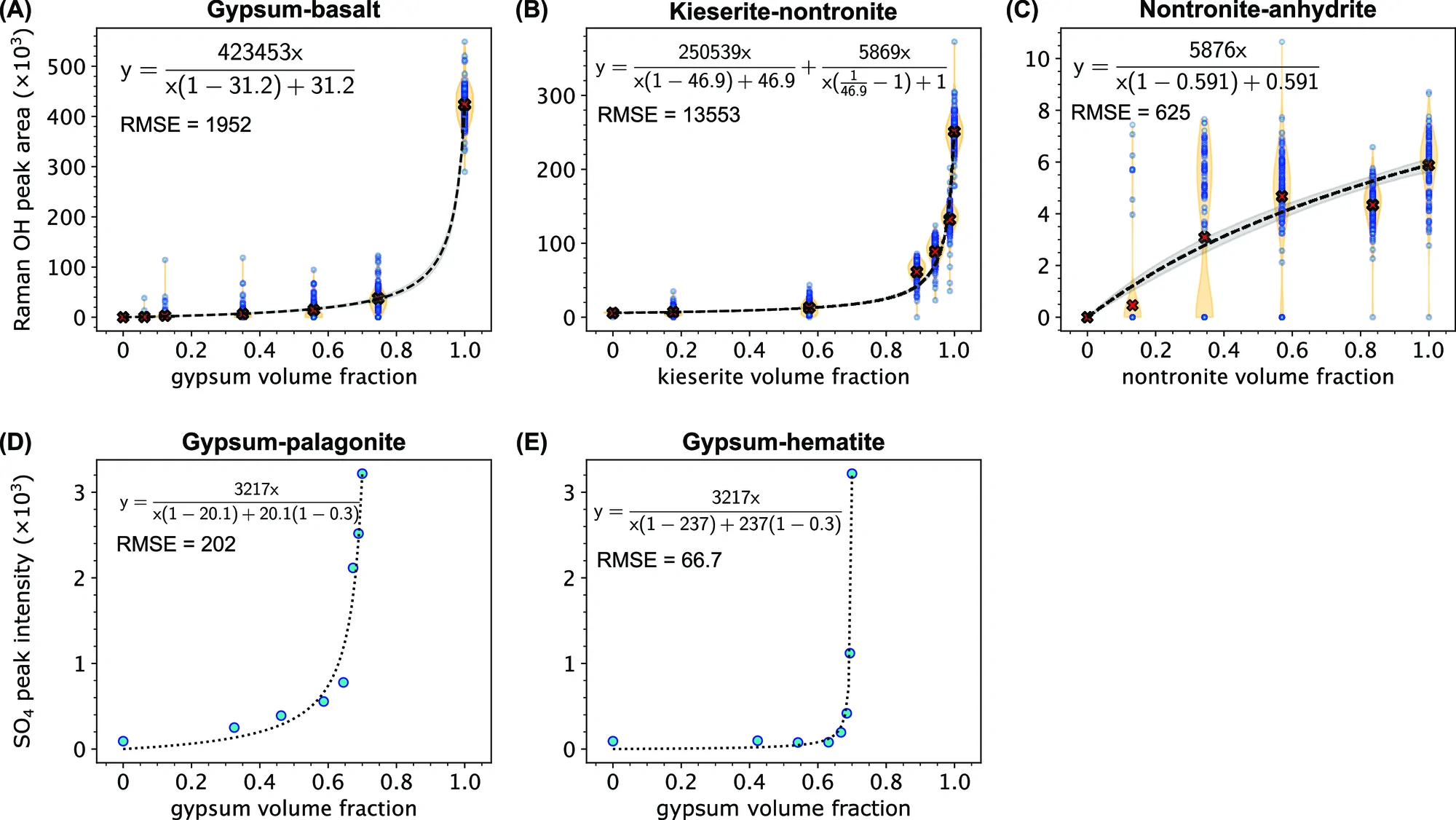
Raman OH peak area as a function of concentration of the hydrated component for five types of low absorptivity + high absorptivity mixtures: (A) gypsum-basalt pressed pellets, (B) kieserite-nontronite pressed pellets, (C) nontronite-anhydrite pressed pellets, (D) gypsum-palagonite powders, digitized from Haney et al.,^48^ (E) gypsum-hematite powders, digitized from Haney et al.^48^ Symbols and colors as in Figure 5. For (D) and (E), only one value of sulfate peak intensity was reported for each concentration of gypsum hence the confidence intervals of the fit parameters are not derived. The root-mean-square-error (RMSE) of the fit is reported in the figure.

To understand the different mathematical forms of the relationship between peak area and concentration for low absorptivity + high absorptivity materials, we rearrange eq 6 using the constraints provided by the measurable average OH peak area of the pure components and the relation *C*_*x*,vol_ + *C*_*y*,vol_ = 1 for a two-component mixture. When *C*_*x*,vol_ = 1, *C*_*y*,vol_ = 0:

11 $$I_{x=1}=\frac{I_{0}\sigma_{x}}{\varepsilon_{0,x}+\varepsilon_{R,x}}=F$$

where *F* is a constant. When *C*_*x*,vol_ = 0, *C*_*y*,vol_ = 1:

12 $$I_{x=0}=\frac{I_{0}\sigma_{y}}{\varepsilon_{0,y}+\varepsilon_{R,y}}=G$$

where *G* is a constant. Using eqs 11 and 12 and eq 8 to express concentration in terms of vol %, eq 6 can then be expressed as

13 $$I=\frac{FC_{x,\mathrm{vol}}}{C_{x,\mathrm{vol}}\left(1-H\right)+H}+\frac{G{(1-C}_{x,\mathrm{vol}})}{C_{x,\mathrm{vol}}\left(\frac{1}{H}-1\right)+1}$$

where $H=\frac{{{(\varepsilon }_{0,y}+\varepsilon }_{R,y})\rho_{y}a_{x}}{{(\varepsilon_{0,x}+\varepsilon }_{R,x})a_{y}\rho_{x}}$ and is a constant.

When only one of the components, *x*, is hydrated and component *y* is anhydrous and does not contribute to the OH peak, σ_*y*_ = 0 and *G* = 0 such that eq 13 becomes

14 $$I=\frac{F\times C_{x,\mathrm{vol}}}{C_{x,\mathrm{vol}}\left(1-H\right)+H}$$

For gypsum-basalt and nontronite-anhydrite, where only one component is hydrated, the average OH peak area as a function of concentration can be modeled with eq 14 (Figure 6(A),(C)). The average OH peak area as a function of concentration for kieserite-nontronite, where both components are hydrated, can be modeled with eq 13 (Figure 6(B)).

The concavity of the average OH peak area as a function of concentration depends on the relative absorptivities of the two components. For example, the concavity for nontronite-anhydrite is concave down because the absorptivity of nontronite, which is the hydrated component taken to be component *x*, has a higher absorptivity than anhydrite, taken to be component *y*. This results in (ε_0,*y*_ + ε_*R*,*y*_) < (ε_0,*x*_ + ε_*R*,*x*_) and consequently *H* < 1 such that the coefficient of *C*_*x*_ is positive. In contrast, for gypsum-basalt and kieserite-nontronite, where the absorptivity of the hydrated component *x* is smaller than component *y*, *H* > 1 such that the coefficient of *C*_*x*_ is negative and the concavity is concave up.

For the gypsum-palagonite and gypsum-hematite powdered mixtures from Haney et al.^48^ that likely have substantial porosity, to model the sulfate peak intensity as a function of concentration, eq 6 needs to be modified to account for the porosity using the relation *C*_*x*,vol_ + *C*_*y*,vol_ + *p* = 1:

15 $$I=\frac{F\times C_{x,\mathrm{vol}}}{C_{x,\mathrm{vol}}\left(1-H\right)+H\left(1-p\right)}$$

where *p* is the porosity. Figure 6(D),(E) show that our model, modified to account for porosity (eq 15), is also able to model the sulfate peak intensity as a function of concentration for the gypsum-palagonite and gypsum-hematite powdered mixtures from Haney et al.^48^ Thus, our models, which are developed based on a theoretical understanding of the effects of sample absorption and Raman scattering, are not instrument or sample-specific. Our models can be applied to any sets of samples measured under any conditions as long as the measuring conditions are consistent within that set of samples.

### High Absorptivity + High Absorptivity Mixtures

We investigated two types of mixtures for the high absorptivity + high absorptivity mixtures, for which the Raman intensity can be characterized by eq 6: nontronite-basalt and serpentine-basalt. Both of these mixtures consist of only one hydrated component, and the average OH peak areas increase with increasing concentrations of the hydrated component (Figure 7).

**Figure 7:**
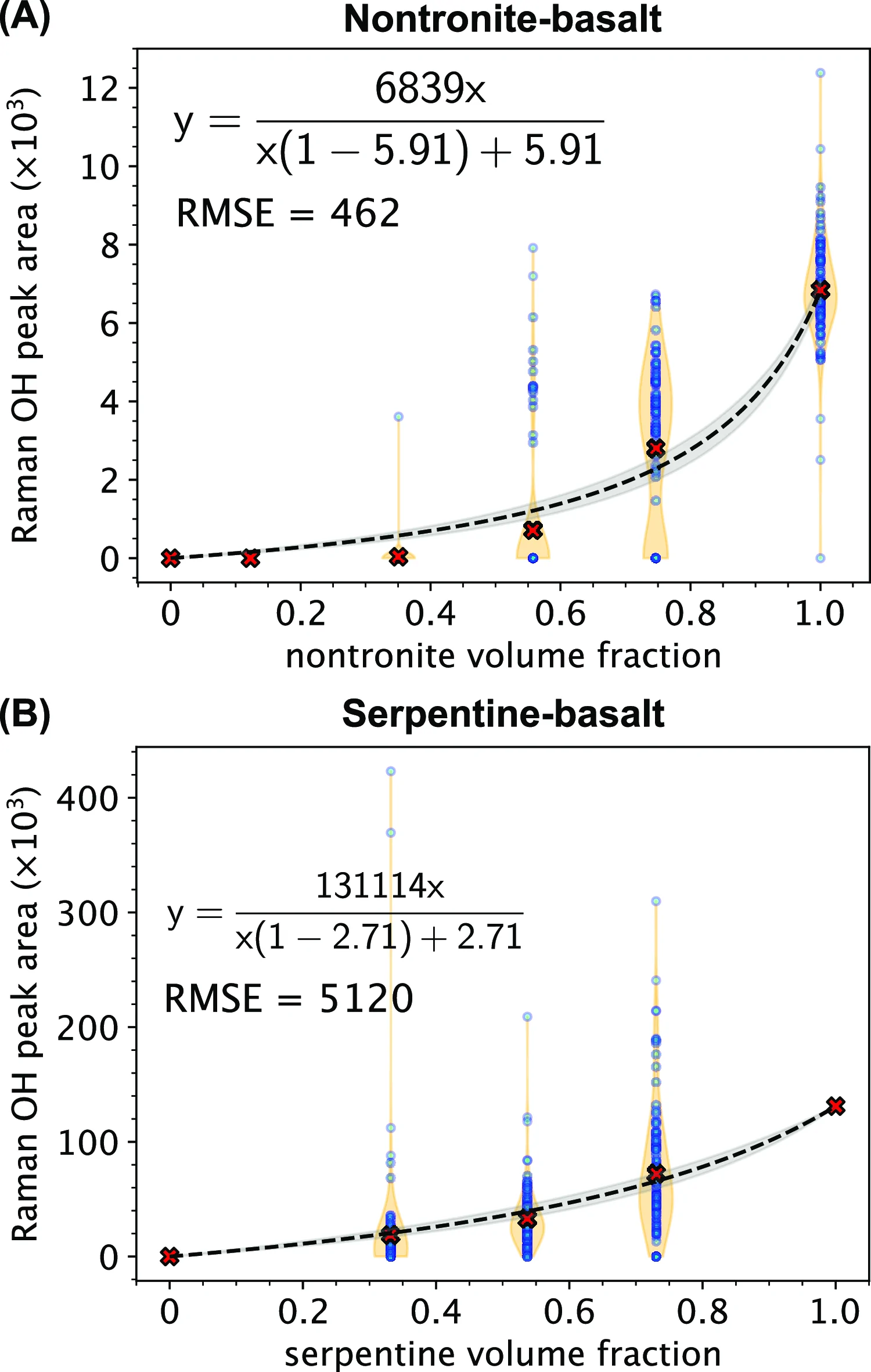
Raman OH peak area as a function of concentration of the hydrated component for two types of high absorptivity + high absorptivity mixtures: (A) nontronite-basalt pressed pellet, (B) serpentine-basalt pressed pellets. Symbols and colors as in Figure 5. The root-mean-square-error (RMSE) of the fit is reported in the figure.

The average OH peak area as a function of concentration for both of these mixtures can be modeled with eq 14. While both of these mixtures are composed of two high absorptivity components, the concave-up curves suggest that the absorptivities of the hydrated components, which are the phyllosilicates nontronite and serpentine, are smaller than the absorptivity of basalt.

## Discussion

### Observed Detection Thresholds and the Dominant Effect of High Absorptivity Materials

The dominant effects of high-absorptivity materials are observed when comparing the Raman peak intensity of the same material mixed in a low or high absorptivity matrix. For example, when kieserite, a low-absorptivity material, is mixed with anhydrite, another low-absorptivity material, at our lowest measured concentration of 22 vol % kieserite, the Raman OH peak area recorded was ∼37000 (Figure 5(A)). In contrast, when kieserite is mixed with nontronite, a high-absorptivity material, 87 vol % of kieserite is needed to produce an equivalent Raman OH peak area of ∼37000 (Figure 6(B)). Similarly, the Raman OH peak of nontronite, a high-absorptivity material, was detectable at 13 vol % mixed in low-absorptivity anhydrite (Figure 6(C)), but this concentration of nontronite in nontronite-basalt was insufficient to produce OH peaks that pass the signal detection threshold, which requires ∼35 vol % nontronite in basalt (Figure 7(A)). When the signal detection threshold is instead set to SNR > 3, the detection threshold of nontronite in anhydrite remains at 13 vol % but is increased to 56 vol % for nontronite in basalt. The trend of requiring higher concentration of a phase for its detection in a dark matrix is consistent regardless of the exact signal detection threshold chosen. Therefore, these results support our qualitative understanding that dark minerals can create challenges in the detection of Raman peaks of materials of both low and high absorptivities due to more significant attenuation.

In the context of Earth and planetary sciences, these results imply that a mineral with low absorptivity such as evaporative salts will need to be present in higher concentration when mixed with high-absorptivity minerals such as Fe-bearing silicate rocks compared to other low-absorptivity minerals. Similarly, minerals of high absorptivity and/or low Raman scattering cross sections such as phyllosilicates need to be nearly pure or mixed with low absorptivity minerals such as evaporative salts in the field of view of the laser to produce observable Raman signatures with field Raman spectrometers such as SHERLOC on the Mars 2020 rover. However, phyllosilicate minerals tend to be dispersed as micrometer-scale alteration products in/around silicate or oxide mineral grains in a matrix and are rarely present as filled veins or vugs that are pure phyllosilicates. Their tendencies to form in dispersed textures could explain the lack of detections of phyllosilicates by the SHERLOC Raman spectrometer relative to widespread detections by orbital and in situ near-infrared spectroscopy.^49–51^

### How Mathematical Relationships Inform Absorptivity of High Absorptivity Materials and Raman Sensing Depths of Low Absorptivity Materials

In “Experimental Verification of Model As Relevant to Geologic Materials”, we have verified our parameterizations of the Raman intensity as a function of concentration through laboratory Raman measurements by treating the unknowns, including Raman scattering cross-section, absorptivity and Raman sensing depth, as constants that are within empirical constants fit by data. We can explore what the parameters obtained from the model fits to the laboratory measurements can tell us about the Raman scattering phenomenon.

In the high absorptivity models, the Raman intensity is independent of the Raman sensing depth (eq 6). This is a direct outcome of our limiting case whereby we assumed that *e* ^–*z*_max_((ε_0,*x*_+ε_*R*,*x*_)*C*_*x*_+(ε_0,*y*_+ε_*R*,*y*_)*C*_*y*_)^ ≪ 1. The physical meaning of this is that a negligible quantity of photons from the sensed volume returns to the detector because photons are almost entirely attenuated within the Raman sensing depth, incoming or outgoing. Thus, the Raman intensity is parameterized by the Raman scattering cross-section and the absorptivity in the high absorptivity models and the sensing depth variation is unimportant (eq 6). One of the fit parameters, $H=\frac{{(\varepsilon }_{0,y}+\varepsilon_{R,y})\rho_{y}a_{x}}{(\varepsilon_{0,x}+\varepsilon_{R,x})a_{y}\rho_{x}}$ (eqs 13 and 14) is solely a function of absorptivity, which allows us to derive the absorptivity of one component when the absorptivity of the other component is known from prior measurements.

On the other hand, in the low absorptivity + low absorptivity model, absorptivity of the materials is unimportant, and the Raman intensity is a function of the Raman sensing depth, in addition to both of the Raman scattering cross sections (eq 9). One could be calculated from knowledge of the other and the empirical fit of our models. Notably, however, neither the Raman sensing depth nor the Raman scattering cross-section is well-characterized for materials of diverse composition and crystallinity. The latter is generally small at orders of ∼10^–30^ cm^2^ molecule^–1^ str^–1^.^21,25^

In both the low absorptivity and high absorptivity models, another unknown of the process of converting empirical constants to SI units is the spectrometer response function, which is the conversion between the intensity of the incident laser, *I*_0_, given in units of W m^–2^, and the photon counts measured by the spectrometer, from which all fit parameters are derived. It is challenging to experimentally or theoretically determine the spectrometer response. A common method to remove the effects of an unknown variable is to evaluate the ratio. We illustrate how to use both the low absorptivity and high absorptivity models in combination to remove the effects of the incident intensity, *I*_0_, and Raman scattering cross sections and estimate the Raman sensing depth *z*_max_. To compare with previous works that have experimentally derived the Raman scattering cross-section of a sulfate peak (see “How Mathematical Relationships Inform Raman Scattering Cross Sections”), we conduct a similar analysis as in “Results and Discussion” but here focused on the sulfate peak intensity instead of the hydration peaks (Figure 8), due to the greater availability of literature data for the sulfate peak. Here, we fit the sulfate peak with corresponding detections of hydration peaks as in “Low Absorptivity + Low Absorptivity Mixtures”, using a Lorentzian function for the SO_4_^2–^ tetrahedra in gypsum that are arranged in a well-ordered crystalline lattice (see “Acquisition and Processing of Raman Spectra”).

**Figure 8:**
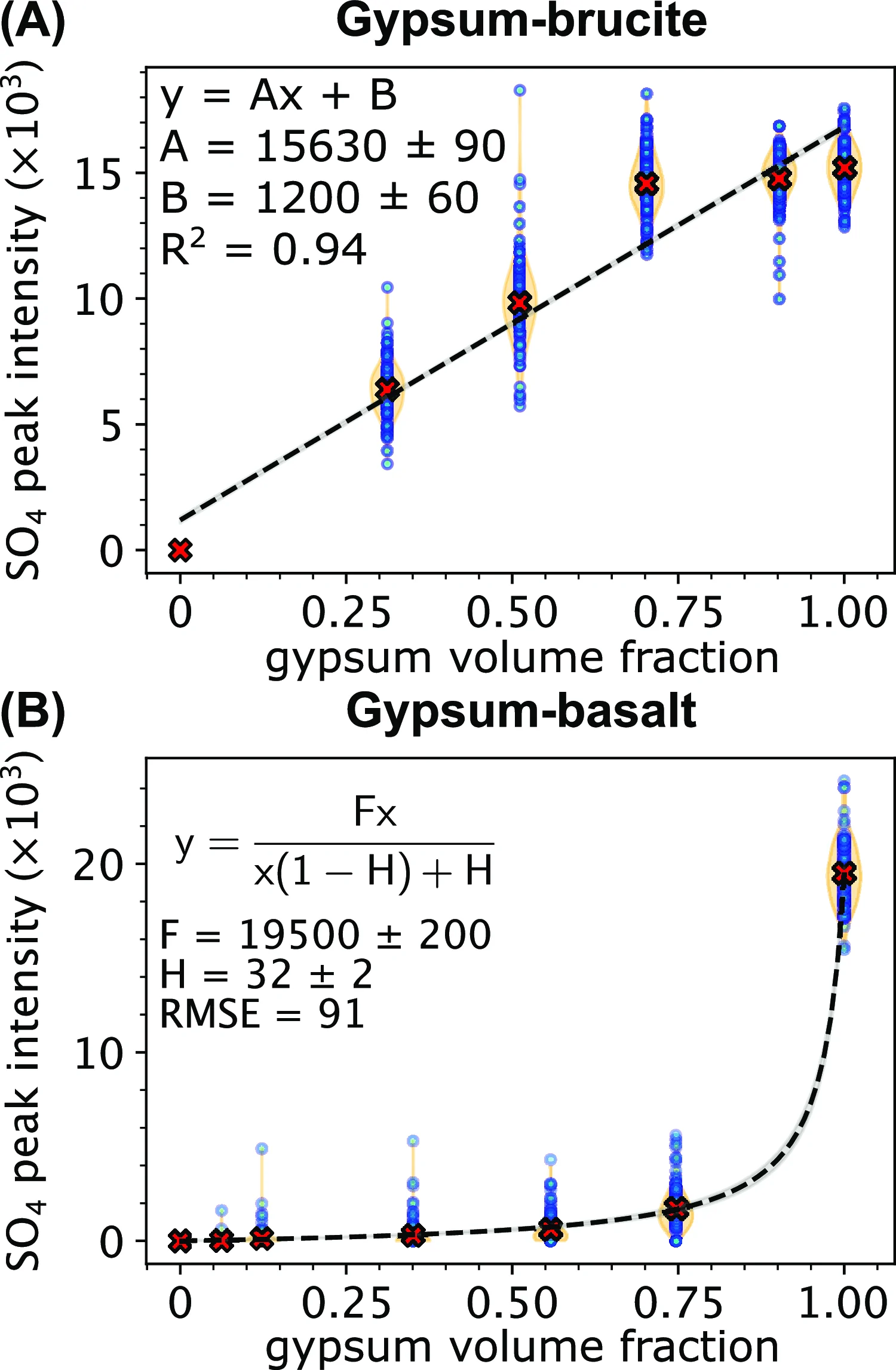
Sulfate peak intensity as a function of gypsum concentration for (A) a low absorptivity + low absorptivity mixture: brucite-gypsum and (B) a high absorptivity + low absorptivity mixture: gypsum-basalt. Symbols and colors as in Figure 5. The root-mean-square-error (RMSE) of the fit to gypsum-basalt is reported in the figure.

The absorption coefficient of basalt, α, is given in Johnson and Grundy^52^ as approximately 110 cm^–1^. Using the relation $\alpha =\varepsilon \frac{\rho }{a}$ and $H=\frac{{(\varepsilon }_{0,y}+\varepsilon_{R,y})\rho_{y}a_{x}}{(\varepsilon_{0,x}+\varepsilon_{R,x})a_{y}\rho_{x}}=\frac{{(\alpha }_{0,y}+\alpha_{R,y})\frac{a_{y}}{\rho_{y}}\rho_{y}a_{x}}{(\varepsilon_{0,x}+\varepsilon_{R,x})a_{y}\rho_{x}}=32\pm 2$ for the gypsum-basalt mixture (Figure 8(B)) where component *x* is gypsum and component *y* is basalt, with ρ_*x*_ = 2.30 g cm^–3^ (Table 1), *a*_*x*_ = 172.17 g mol^–1^, we obtain the absorptivity of gypsum, ε_gyp_, to be 260 ± 20 cm^2^ mol^–1^. We have assumed that ε_0,*x*_ = ε_*R*,*x*_ and ε_0,*y*_ = ε_*R*,*y*_, which is a reasonable assumption as the laser wavelength (514.5 nm) and Raman shift wavelength are similar (∼542.6 nm). Then, we take the ratio between $\frac{I_{0}\sigma_{x}}{\varepsilon_{0,x}+\varepsilon_{R,x}}=F$ , a fit parameter from the high absorptivity model (eqs 11 and 14) for gypsum-basalt, where component *x* is gypsum (Figure 8(B)), and $I_{0}z_{\max}\left(\sigma_{x}\frac{\rho_{x}}{a_{x}}-\sigma_{y}\frac{\rho_{y}}{a_{y}}\right)=A$ , the slope from the linear low absorptivity model (eqs 9 and 10), where component *x* is gypsum and component *y* is brucite (σ_*y*_ = 0; brucite is non-Raman scattering at 1008 cm^–1^ relevant to gypsum; Figure 8(A)), such that

$$\frac{\frac{I_{0}\sigma_{\mathrm{gyp}}}{\varepsilon_{0,\mathrm{gyp}}+\varepsilon_{R,\mathrm{gyp}}}}{I_{0}z_{\max}\sigma_{\mathrm{gyp}}\frac{\rho_{\mathrm{gyp}}}{a_{\mathrm{gyp}}}}=\frac{19500\pm 200}{15630\pm 90}$$

Using ρ_gyp_ = 2.30 g cm^–3^ (Table 1) and *a*_gyp_ = 172.17 g mol^–1^, we can solve for *z*_max_ of gypsum to be 1.15 ± 0.09 mm. Our calculated *z*_max_ for gypsum is fully consistent with the findings of Edwards et al.^35^ who observed Raman signal from a crystalline gypsum sample originating 1 mm but not at 3 mm below the surface. Perfect agreement is not expected as there is likely to be sample-dependent variation in sensing depth. The crystalline gypsum sample used in Edwards et al.,^35^ which is of selenite variety, likely has a higher optical transparency than our powdered, pressed pellet samples, as prior studies have shown scattering loss at the grain boundaries could reduce the effective depth from which Raman signals are observed.^28–33^

The Raman sensing depth *z*_max_ will vary depending on the instrument and sample characteristics, making it difficult to quantify a universal Raman sensing depth for a given mineral. Our discussions above show that we can use the low absorptivity + low absorptivity and high absorptivity + high/low absorptivity models together, with the selection of appropriate samples, to deduce the Raman sensing depth if the absorptivities of some components are known.

### How Mathematical Relationships Inform Raman Scattering Cross Sections

With an estimated value of the Raman sensing depth and/or the absorptivity, in principle, it is possible to estimate the Raman scattering cross sections from the fit parameters of the low absorptivity model and high absorptivity model. However, as described in “How Mathematical Relationships Inform Absorptivity of High Absorptivity Materials and Raman Sensing Depths of Low Absorptivity Materials”, it is challenging to experimentally or theoretically determine the conversion between the intensity of the incident laser, *I*_0_, given in units of W m^–2^, and the photon counts measured by the spectrometer. We illustrate the potential in the fit parameters from our low absorptivity and high absorptivity models for deriving the Raman scattering cross sections of different materials through the following back-of-the-envelope calculation with an assumed value for *I*_0_.

We use the observed mean Raman sulfate peak intensity of pure gypsum (15200 counts; Figure 8(A)) and the low probability of the Raman effect, whereby approximately 1 in 10^8^ incident photons will undergo Raman scattering,^53^ to estimate the incident intensity, *I*_0_ = 15200 × 10^8^ = 1.52 × 10^12^ counts. For the high absorptivity gypsum-basalt mixture,

$$\frac{I_{0}\sigma_{\mathrm{gyp}}}{\varepsilon_{0,\mathrm{gyp}}+\varepsilon_{R,\mathrm{gyp}}}\times N_{A}=\frac{{1.52\times 10^{12}\left[\mathrm{counts}\right]\sigma }_{\mathrm{gyp}}}{260+260\left[{\mathrm{cm}}^{2}{\mathrm{mol}}^{-1}\right]}\times 6\times 10^{23}\left[\mathrm{molec}{\mathrm{mol}}^{-1}\right]=19500\left[\mathrm{counts}\right]$$

We have included the units in parentheses in the equation for consistency purposes and introduced an additional product with Avogadro’s number, *N*_A_, to convert from moles to number of molecules. Solving for the Raman scattering cross-section of gypsum, we obtain σ_gyp_ = (1.11 ± 0.09) × 10^–29^ cm^2^ molecule^–1^.

For the low-absorptivity gypsum-brucite mixture,

$$I_{0}z_{\max}\sigma_{\mathrm{gyp}}\frac{\rho_{\mathrm{gyp}}}{a_{\mathrm{gyp}}}\times N_{A}={1.52\times 10^{12}\left[\mathrm{counts}\right]\times 0.115\left[\mathrm{cm}\right]\times \sigma }_{\mathrm{gyp}}\times \frac{2.3\left[g{\mathrm{cm}}^{-3}\right]}{172.17\left[g{\mathrm{mol}}^{-1}\right]}\times 6\times 10^{23}\left[\mathrm{molec}{\mathrm{mol}}^{-1}\right]=15630\left[\mathrm{counts}\right]$$

We similarly obtain *σ*_gyp_ = (1.12 ± 0.09) × 10^–29^ cm^2^ molecule^–1^ as the Raman scattering cross-section of gypsum. The consistency in the Raman scattering cross-section of gypsum derived from the low absorptivity model and high absorptivity model gives us confidence that our models are accurately reflecting the optical phenomena of absorption and Raman scattering.

Dudik et al.^54^ experimentally derived a Raman scattering cross-section of the sulfate peak for an aqueous Na-sulfate solution as 9.9 × 10^–30^ cm^2^ molecule^–1^ str^–1^. However, we emphasize that a direct one-to-one comparison between our Raman scattering cross-section and that of Dudik et al.^54^ is not possible because of our uncalibrated estimate for *I*_0_ from counts (see above discussion) that does not take into account the instrument and spectrometer-dependent properties such as the illuminated area, acquisition time, collection solid angle, optical throughput, spectrometer response, detector quantum efficiency, and gain. Nevertheless, this exercise illustrates that the low and high absorptivity models we have developed in this work, combined with a reasonable understanding of the conversion between incident laser flux density and spectrometer counts, a parameter that could be reported by manufacturers to enable such calculations, can provide a pathway to experimentally deriving the Raman scattering cross-section for many minerals and other materials.

Optimal design and operation of Raman spectrometers to be used in field settings benefit from the ability to quantitatively calculate limits of detection for constituents of interest. In the field of geological and planetary sciences, being able to quantify the abundances of different minerals is significant for understanding the formation processes and conditions of past geological environments. The estimates of Raman sensing depth and Raman scattering cross sections from application of our models using eqs 5 and 6, coupled with a calibration to convert between laser intensity and spectrometer counts can provide the forward path for achieving quantitative Raman spectroscopy in field settings.

### Towards an Empirical Method for In Situ Mineral Quantification from Raman Spectroscopy

We can quantify the concentration of a given material in a mixture sample from Raman peak intensities if we know the Raman sensing depth, Raman scattering cross sections, and absorptivity of constituents (“How Mathematical Relationships Inform Absorptivity of High Absorptivity Materials and Raman Sensing Depths of Low Absorptivity Materials” and “How Mathematical Relationships Inform Raman Scattering Cross Sections”). However, these quantities are not currently well-characterized for materials of diverse composition and crystallinity. Thus, in planetary or other field settings where we do not know a priori what minerals are present, other empirical methods are required.

We illustrate how our models, Raman mapping RGB images, and independent quantitative compositional constraints can be used to estimate the lowest concentration at which Raman features are detectable in a case study of the ‘Dourbes’ sample measured by the SHERLOC Raman spectrometer on Mars. SHERLOC measured four different areas in Dourbes for a total of 400 scan points of Raman spectra (Figure 9(A)) and collected higher-resolution grayscale images by the Autofocus Context Imager (ACI) that provide the context for the locations of the Raman spectra. The minerals identified by SHERLOC Raman spectroscopy in Dourbes include olivine, an unidentified silicate group mineral, perchlorate and/or phosphate, pyroxene, hydrated Mg-sulfate and carbonate.^7,8,10^

**Figure 9:**
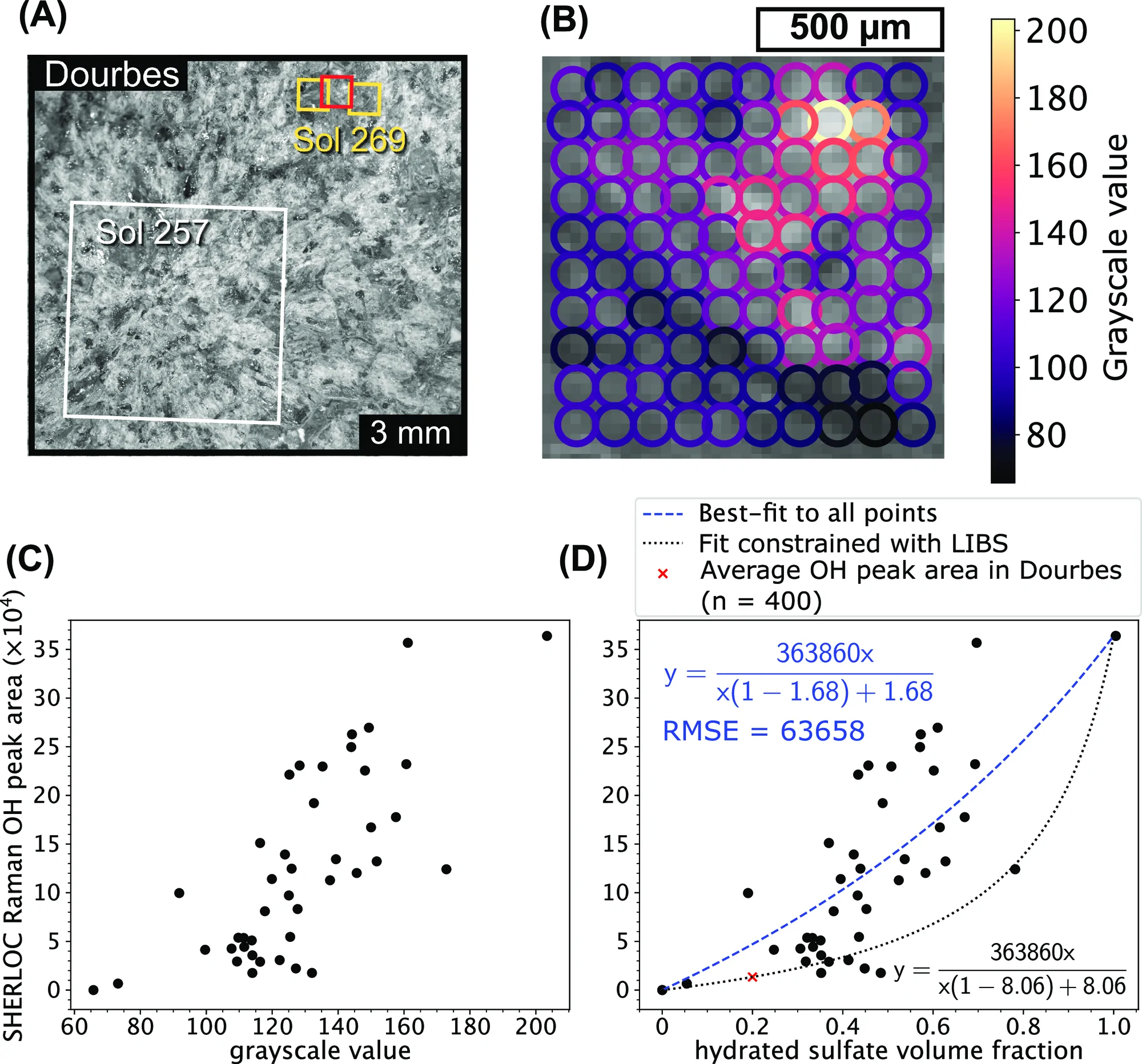
(A) The footprints of SHERLOC scans for the abraded target Dourbes. The image was acquired with the Autofocus Context Imager (ACI) at grayscale. (B) The grayscale values were calculated for each SHERLOC laser scan point for the sol 269 Dourbes detail 1 scan, which is marked by a red square in (A). The SHERLOC Raman OH peak area measured in the sol 269 Dourbes detail 1 scan^8^ as a function of (C) grayscale value and (D) the hydrated sulfate concentration, which was derived assuming a linear relation with grayscale value. The average OH peak area from all 400 scan points in Dourbes is marked by a red cross. The dashed line is the best-fit to all points while the dotted line is the fit using the quantitative constraint of hydrated sulfate abundance estimated from LIBS.

We focus on the ‘sol 269 Dourbes detail 1’ scan, which has the highest number of hydrated sulfate detections^8,10^ and is marked by a red square in Figure 9 (A). A visual examination of this region suggests it can be classified as a mixture made up of a low absorptivity component that is the hydrated sulfate and one or more higher absorptivity component(s) (Figure 9(B)), even though the SHERLOC laser wavelength at 248.6 nm is different than the visible wavelength range that the ACI operates in. For simplicity, we assume that points in Dourbes with hydrated sulfate detections are made up of two components: hydrated sulfate and a high-absorptivity, could be a silicate material that produces no Raman peaks in the SHERLOC spectral range of 800-4000 cm^–1^. We calculated the grayscale values for each SHERLOC laser scan point from the ACI image, using the OpenCV library of Python.^55^ The SHERLOC laser beam is in the shape of an annulus with a diameter of ∼100 μm but in our calculation of the grayscale values, we included all the pixels contained within a circle of diameter ∼ 100 μm.

As we do not have independent constraints on how the concentration of the hydrated sulfate relates to the grayscale value, we make three simple assumption: (1) this relationship is linear, (2) the largest grayscale value of 203, which is also the point with the largest SHERLOC Raman OH peak area, is comprised of pure hydrated sulfate (100 vol %), and (3) the point with no Raman mineralogical detections and the lowest grayscale value of 66 does not contain any hydrated sulfate (0 vol %). The observed SHERLOC Raman OH peak area (from Phua et al.^8^)­as a function of grayscale value (Figure 9(C)) and estimated concentration of hydrated sulfate (Figure 9(D); black points) show some scatter. The best-fit low absorptivity + high absorptivity model (Figure 9(D); blue dashed curve) has a high RMSE implying it does not fit the data well, even though the concave up fit is consistent with the hydrated sulfate having a lower absorptivity than the silicate matrix in which it is mixed in (see “Low Absorptivity + High Absorptivity Mixtures”). In this fit, the lowest concentration at which the Raman OH peaks of hydrated sulfates were detected at Dourbes with SHERLOC is 3.0 vol %.

Next, we incorporate constraints from other quantitative instruments. For the Mars 2020 rover, the SuperCam laser-induced breakdown spectrometer (LIBS) provides a quantitative estimate for the bulk water content of solid surfaces using the peak area of the 656 nm H emission line normalized to oxygen.^8,56^ Phua et al.^8^ reported the mean LIBS H peak area for Dourbes to be 0.046. Using the calibration curves that have been developed for ChemCam by Rapin et al.^57^ and Thomas et al.,^39^ this H peak area corresponds to water content of 5.4 wt %. Assuming that this water is all in sulfate, this corresponds to 14.4 wt % (approximately 20 vol %, assuming the rest of the material is silicate of density 3 g cm^–3^) of hydrated sulfate, calculated for MgSO_4_·4H_2_O, which is an average number of water per formula unit reported in Phua et al.^8^ and Siljeström et al.^10^ We calculated the average OH peak area for Dourbes using all 400 SHERLOC scan points to cover an area more comparable to the LIBS measurements and fit for the Raman intensity as a function of hydrated sulfate abundance using the LIBS-derived constraint, which is that the average Raman OH peak area for Dourbes corresponds to a hydrated sulfate concentration of 20 vol % (Figure 9(D)). In this fit (Figure 9(D); black dotted curve), the lowest concentration at which the Raman OH peaks of hydrated sulfates were detected at Dourbes with SHERLOC is 10.9 vol %.

While the results of color analyses from RGB images and LIBS quantification are not entirely consistent with each other, it is encouraging that the lowest concentration for detectable Raman OH peaks from these two independent methods differs only over 3-10.9 vol %. Dourbes – and many rocks on Mars – are complex, multi-mineralic systems so the deviation from the two-component mixing assumed here is unsurprising; in fact, there may be both a linear and nonlinear relationship from multi-endmember mixing (Figure 9(D)). Furthermore, color is often not linear with amount of component. To assess the sensitivity of the derived detection threshold to the functional relationship between color and concentration that we have assumed, we also modeled concentration as a square root function of color using assumptions (2) and (3) from above: $\mathrm{concentration}=8.5\sqrt{\mathrm{color}-66}$ . We find that the lowest concentration for detectable Raman OH peaks using this square root function is 7.1 vol %, which is within the range of 3–10.9 vol % based on the linear function. Nevertheless, we note that the range of 3–10.9 vol % is merely a first order preliminary approximation and the confidence of using RGB images as a proxy for the concentration of hydrated sulfates can be improved by knowing functionally how the color scales as a function of concentration. This can be done by measuring some standards of expected geological mixtures, whether in the lab under similar illumination conditions or having these standards as calibration targets alongside the instrument.

It is also possible to characterize the sample absorptivity by reflectance spectroscopy *in-situ*. This is a possibility that can be explored for the MMX rover, which will be equipped with a reflectance spectrometer in addition to a Raman spectrometer.^4^ An estimation of the sample absorptivity enables calculation of sensing depth (see “How Mathematical Relationships Inform Absorptivity of High Absorptivity Materials and Raman Sensing Depths of Low Absorptivity Materials”), which can provide useful context for understanding the stability of hydrated minerals and organic matter through their detections or non-detections. In addition, knowing the Raman sensing depth can also help us understand discrepancies in observations across different instruments. There is thus potential for a relatively straightforward way of developing an *in-situ* calibration model that enables quantitative analyses from Raman spectroscopy.

## Conclusion

We developed mathematical models that quantitatively explain Raman peak intensity as a function of concentration, Raman scattering cross section, absorptivity, and the Raman sensing depth, all of which are sample- and wavelength-dependent properties. Our analysis revealed different functional forms for two-component mixtures, depending on the relative absorptivities of the materials and whether one or both materials have the Raman peak of interest. We verified the performance of the mathematical models through laboratory Raman measurements of prepared mixtures for three types of two-component mixtures: (1) low absorptivity + low absorptivity materials, (2) low absorptivity + high absorptivity materials, and (3) high absorptivity + high absorptivity materials. Our systematic exploration of the Raman intensity as a function of concentration for these different mixtures also demonstrates quantitatively that it is challenging to detect minerals when they are mixed in a matrix of high absorptivity minerals.

With knowledge of the Raman scattering cross section and the absorptivity, our models can be used to solve for the abundance of materials through Raman spectroscopy. Conversely, our models potentially provide an alternative way to deduce some of these properties if at least one of these properties has been characterized and is known through other lab measurements. Provision of the instrument response function for a Raman system relating photon counts to irradiance would enable determining Raman cross-section from experiment. Further development of methods that can characterize sample absorptivity concurrent with Raman intensities of the same target material can lead to a higher confidence in the derived abundances and the Raman sensing depth, providing a pathway to field-based Raman quantification of minerals.
